# Correction: The protective effect of propofol on ionizing radiation-induced hematopoietic system damage in mice

**DOI:** 10.1039/c9ra90086a

**Published:** 2019-11-19

**Authors:** Xiaoliang Han, Fengtao Sun, Ying Zhang, Jinyan Wang, Qingguo Liu, Ping Gao, Shubo Zhang

**Affiliations:** Affiliated Hospital, North China University of Science and Technology Tangshan Hebei 063000 China mayastarfx2008@163.com; Tangshan Gongren Hospital Tangshan Hebei 063000 China

## Abstract

Correction for ‘The protective effect of propofol on ionizing radiation-induced hematopoietic system damage in mice’ by Xiaoliang Han *et al.*, *RSC Adv.*, 2019, **9**, 36366–36373.

Fig. 5 as published was actually the same as Fig. 7; the corrected version of the figure (with associated legend) is shown below.

**Fig. 1:**
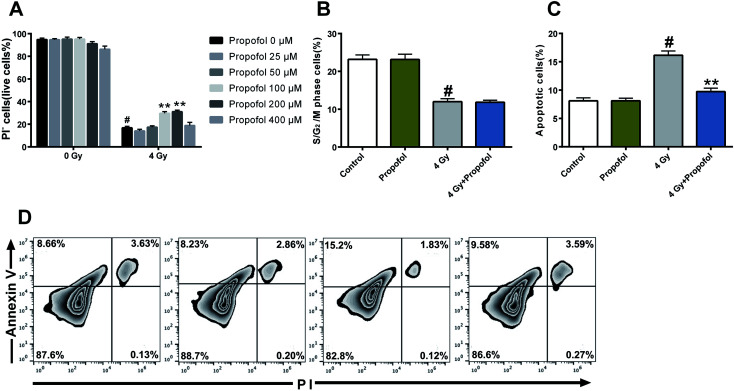
Propofol inhibits IR-induced cell death and apoptosis. Propofol at concentrations of 25 μM, 50 μM, 100 μM, 200 μM, and 400 μM was added to the culture medium 30 min before Lineage^−^ cells were exposed to 4 Gy, and then cell death, apoptosis and cell cycle analyses were performed. (A) The percentage of live cells; (B) the percentage of proliferative (S/G_2_/M phase) cells; (C) the percentage of apoptotic cells; (D) representative flow scatter plots of cell apoptosis. Data are presented as means ± SEM (*n* = 5), #*p* < 0.05 *vs.* control, **p < 0.05 *vs.* 4 Gy.

The Royal Society of Chemistry apologises for these errors and any consequent inconvenience to authors and readers.

## Supplementary Material

